# Community-based training of medical students is associated with malaria prevention and treatment seeking behaviour for children under 5 years in Uganda: a study of MESAU-MEPI COBERS in Uganda

**DOI:** 10.1186/s12909-018-1250-y

**Published:** 2018-06-08

**Authors:** James Henry Obol, Peter Akera, Pamela Atim Ochola, Wilfred Arubaku, Hussein Oria, Kenneth Luryama Moi, Denis Anywar Arony, Kaducu Felix

**Affiliations:** 1 Department of Public Health, Faculty of Medicine, Gulu University, P.O Box 166, Gulu, Uganda; 2 Deaprtment of Dental Surgery, Faculty of Medicine Mbarara University of Science and Technology, P.O Box 1410, Mbarara, Uganda; 3 Department of Pharmacy, School of health Sciences Makerere University, P.O Box 7072, Kampala, Uganda; 4 Department of Medical Microbiology & Immunology, Faculty of Medicine, Gulu University, P.O Box 166, Gulu, Uganda; 5 Department of Biochemistry, Faculty of Medicine, Gulu University, P.O Box 166, Gulu, Uganda

**Keywords:** COBERS, Malaria prevention and treatment seeking behaviour, Children under 5, Uganda

## Abstract

**Background:**

Four university medical training institutions in Uganda have trained students at off-site health facilities under community-based education and Research Service (COBERS) programme for over 5 years. One of the major components of COBERS placement is for the students to provide health education in the communities about malaria as a major public health disease in Uganda. This study seeks to assess if targeted community-based medical education programme is associated with better prevention and treatment seeking behaviours in the management of malaria, a leading cause of morbidity and mortality of children under five in Uganda.

**Methods:**

A cross-sectional survey was done to compare communities around health facilities where medical students were placed at COBERS sites with communities around similar health facilities where medical students were not placed (non-COBERS sites). We randomly selected two villages near each health facility and consecutively selected 10 households per village for interviews using nearest-neighbour method. We used a structured questionnaire to interview household heads on malaria prevention and treatment seeking behaviour for children under 5 years. We performed univariate analysis to determine site and demographic characteristics and performed a multivariate logistic regression to assess association between dependant and independent variables.

**Results:**

Five hundred twenty-three (66.8%) of the children under 5 years in COBERS communities slept under Insecticide Treated Nets (ITNs) the night before survey compared with 1451 (57.8%) in non-COBERS communities (AOR = 0.66, *p* = 0.017). 100 (60.0%) of children under 5 years in COBERS communities sought care for fever within 24 h of onset compared with 268 (47.0%) in non-COBERS communities (AOR = 0.71, *P* = 0.009).

**Conclusion:**

The presence of COBERS in communities is associated with improved malaria prevention and treatment-seeking behaviour for parents of children under 5 years. Further study needs to be done to determine the long-term impact of COBERS training program on malaria control and prevention in Uganda, along with other effects of COBERS.

**Electronic supplementary material:**

The online version of this article (10.1186/s12909-018-1250-y) contains supplementary material, which is available to authorized users.

## Background

Malaria is a threat to the lives of about 3.2 billion people globally [1]. Children under five years are particularly vulnerable to severe disease and death when infected with malaria in endemic areas [2]. In Africa, malaria constitutes 10% of the disease burden and is the leading cause of under-5 mortality accounting for about 20% [3]. Uganda has the third highest number of deaths attributable to malaria in Africa and has some of the highest recorded malaria transmission rates [4]. Malaria in Uganda constitutes a major public health problem responsible for between 30 and 50% of all outpatient consultations and an estimated 35% of hospital admissions [5]. Malaria and malaria related-illnesses contributed to between 20 to 23% of deaths among children aged less than five years in Uganda [6]. The 2009 Uganda Malaria Indicator Survey showed that 52% of children under 5 years had malaria as determined by rapid diagnostic blood testing [7] while the 2014–15 Uganda Malaria Indicator Survey (UMIS) shows that overall, 31% of children had a fever in the two weeks preceding the survey [malaria indicator survey]. The 2014–15 UMIS also shows an improvement in testing for malaria at 36% compared with 17% in 2009 [8].

Prompt access to effective malaria treatment is vital for the success of malaria control worldwide. The Roll Back Malaria (RBM) partnership set a target by the year 2010 of ensuring that 80% of those suffering from malaria have prompt access to, and can correctly use, affordable and appropriate treatment within 24 h of the onset of symptoms [9]. However, many African countries are far below these targets, with only a few of the fevers being treated promptly and effectively [10, 11]. The 2014–15 UMIS also shows slight differences between rural and urban in the Percentages of under 5 children who took any Artemisinin-based Combination Therapy (ACT) same or next day at 40.2 for urban and 50.2 for rural [8].

The barriers to prompt and effective treatment for malaria among children under 5 in Uganda include: limited access to adequate treatment for malaria or fever, increasing resistance of malaria parasites to medicines, incorrect or inadequate malaria treatment at home or within communities [6] and fewer children with fever taking an antimalarial drug within the recommended time frame [4].

Insecticide-treated nets (ITNs) are effective in reducing malaria-related morbidity and mortality and because of this they are increasingly being utilized in sub-Saharan Africa and other malaria-endemic areas [12]. Despite this, only 43% of Ugandan children under the age of 5 years slept under an ITN the night before a national survey as noted in the Uganda Demographic and Health Survey (UDHS) report [4]. Barriers to malaria prevention among children under five in Uganda include low coverage of indoor residual spraying (IRS) and inadequate utilization of ITNs with only 46.5% of children reported to have slept under ITN or in a house sprayed with IRS thereby undermining efforts to control malaria among this vulnerable population [4]. However, in the 2014–15 UMIS shows an improvement in the use of ITN by children under 5 with the percentage who slept under an ITN the night before the survey was 74% compared with 33% reported in the 2009 UMIS [8].

Given the severity of the malaria conditions in Uganda, there is need to explore innovative ways of improving utilization of effective interventions in prevention and treatment of malaria in communities. Training of health professionals at community level has been conceived as a sustainable initiative to address this gap. A consortium of undergraduate medical training universities in Uganda (Makerere University, Gulu University, Mbarara University of Science & Technology and Kampala International University) formed the Medical Education for Equitable Services to All Ugandans (MESAU) in 2010, with funding from the NIH & Fogarty International Centre under Medical Education Partnership Initiative (MEPI) and technical support from Johns Hopkins University and Case Western Reserve University.

All the Uganda MESAU institutions have trained their medical and other health professions students in community-based education research and service (COBERS) as a compulsory part of their health professions curricula for the last five years. Although each institution implements her program differently, the training involves having students participate in various primary care activities including health education and health promotion, immunisation, family planning counselling, growth monitoring and community outreach during their placements.

The COBERS is a modified version of Community-based education (CBE) which involves the integration of education and practice in the community within the learning process [13]. The increasing need to address the universal right to health and contributes to the “Health for All” strategy through promotion of primary health care (PHC) led to the integration of CBE into training curricula of many health professional training institutions in the world from the 1990s [14]. Results from studies done in Indonesia [15], Nigeria [16] and Uganda [17] indicate that community members appreciate CBE and believe that the students’ activities were valuable.

The COBERS training is intended to have students develop competencies in dealing with common health conditions found at community level in Uganda, as well as contribute to improve health in these communities through research and services. During COBERS placement, the students get involved in Primary Health Care (PHC) activities like community mobilisation, home and school visits and health educational talks. Since malaria is the leading cause of morbidity and mortality in Uganda, all COBERS students provide health education on malaria prevention and treatment to patients at the health centres and during community outreaches.

MESAU is assessing the effectiveness and relevance of COBERS to learn lessons to improve the program, and to establish the credibility of COBERS to stakeholders, in part by identifying the broader impact of COBERS on communities. We report here part of the baseline study in which we compared malaria prevention and treatment-seeking behaviour for children under 5 years between communities near COBERS health facilities and non-COBERS communities.

## Methods

### Design and setting

This cross sectional analytical study assessed malaria prevention and treatment seeking behaviours for children under 5 years among communities who live near health facilities in Uganda. We compared communities near health facilities that have been used for student placements during COBERS (COBERS sites) with communities near health facilities that have not been used for COBERS (non-COBERS sites). COBERS placement range from 1st to 4th year of study and all the universities have a module on environmental health and disease control in their programs. This module prepares the students to engage with the community during health education and health promotion activity which they undertake during their COBERS placement. The COBERS placements last between 4 and 8 weeks at the health facilities with students who are in their 4th year of study spent more time providing curative and health promotion activity in the community. In Uganda, medical training institutions admit all qualified students from all over the country based on their choices and therefore there is regional representation in all the medical training institutions in Uganda. The COBERS sites were chosen by the medical training institutions base on geographical location of the medical training institutions which enable them to provide supervision of the medical students during their COBERS placement. The study population consisted of heads of households or adult members of the households who were knowledgeable about the health of all members living in the households within the study villages.

### Sampling

Each institution had its own sampling frame consisting of at least twice the number of health facilities that the institution was likely to use over the next four years from 2011/12 academic year, but which had never been used for COBERS. The health facilities in the sampling frame were those within the geographical area that the institution was willing to send students to and should have met the minimum requirements established by MESAU, namely (i) administration and management willing to support the students; (ii) availability and willingness of qualified site tutors/preceptors; (iii) outpatients department in place; (iv) community outreach program in practice and (v) safe accommodation for students. In addition to the geographical area, each institution’s health facilities were matched by level (HCIII, HCIV and District Hospital). The two facilities that were closest to each other in location and had the same level were paired, and one facility was randomly assigned to be used by students starting in the 2011/2012 academic year (intervention site) and the other to be used a comparison site. A health facility would not be an intervention site for one institution and a comparison site for another and neither would health facilities be intervention or comparison sites for more than one institution. These sites were selected purposively first by eliminating any Regional Referral Hospitals and any shared sites. The health facilities were then stratified into 10 regions as per UDHS report [4] and random selection within each region and matching to new sites within each region was done to ensure that all regions were represented. Table 1 shows how the health facilities were distributed. Because MESAU institutions had been implementing CBE for at least 5 years prior, a decision was made to add a cross-sectional aspect to the main evaluation, in which health facilities that had been used for CBE would be compared to the new sites selected for the main evaluation. Each institution then randomly selected from amongst the health facilities it uses for COBERS. For this study, these are referred to as COBERS sites, while the newly selected health facilities which will later be used in the post evaluation as (intervention and comparison) are referred to as non-COBERS sites.

**Table 1:** Health facility characteristics

Characteristic	COBERS (*N* = 30)		Non-COBERS (*N* = 90)	
	n	%	n	%
Location				
Rural	27	90.0	73	81.1
Urban	3	10.0	17	18.9
Level				
Hospitals	5	16.7	18	20.0
Health Centre IV	13	43.3	31	34.4
Health Centre III	12	40.0	41	45.6
Location with respect to malaria risk				
High	14	46.7	44	48.9
Medium	5	16.7	16	17.8
Low	11	36.7	30	33.3

### Household survey

A total of 240 villages (60 villages in COBERS and 180 villages in non-COBERS) within 2 h walking distance were randomly selected for the survey from around the 120 health facilities. In each health facility, names of all surrounding villages were written down and two villages were randomly selected for the survey. In each village, ten households were selected for interview after randomly selecting a starting point and using the nearest-next door method. All the households that had a child under five years were eligible for the survey. All households’ heads or adult members of the households who were knowledgeable about the health of all members living in the households were interviewed about malaria prevention and treatment among children under 5 years using a structured questionnaire adopted from Demographic and Health Survey (DHS) (Additional file 1) between August 2012 and January 2013.

### Variables

Our dependent variables were ITN use by under 5 years children in the previous night, experiencing fever among under 5 years children in last two weeks before the survey, treatment seeking for fever among under 5 years within one day of fever onset, and taking antimalarial drug within 24 h of fever onset. The independent variables were COBERS status (COBERS vs non-COBERS), household wealth quintile, sex of the child, household location (urban versus rural), and malaria risk zoning according to Uganda Bureau of Statistics [7].

### Data analysis

Data was analysed using Stata 12 (StataCorp, College Station, Texas 77,845 USA). Univariate analysis was performed to determine site and demographic characteristics while the logistic regression was used to assess association between dependant and independent variables. We used principal component analysis method [18] to generate household wealth quintile base on household assets. This is because getting information on income and expenditure for household is not feasible among majority of Ugandans who lives in rural areas [18]. Data with multiple responses were recorded into binary variables [18]. The following socioeconomic indicator variables were used to classify the household economic status: Floor (Natural floor, Finished floor), Roof (Natural roofing, Finished roofing), Walls (Natural walls, Finished walls), Fuel for cooking (Electricity/LPG/Natural gas/Biogas/Kerosene/paraffin/Charcoal, Firewood/Straw/shrubs/grass/Animal dung), Room for cooking (Yes, No), Have kitchen (Yes, No), Have chimney (Yes, No), Electricity (Yes, No), Radio (Yes, No), Cassette player (Yes, No), Television (Yes, No), Mobile phone (Yes, No), Fixed telephone (Yes, No), Refrigerator (Yes, No), Table (Yes, No), Chair (Yes, No), Bed (Yes, No), Clock (Yes, No), Bicycle (Yes, No), Motorcycle/Scooter (Yes, No), Owning land for agriculture (Yes, No), Animal drawn-cart (Yes, No), Local cattle (Yes, No), Exotic cattle (Yes, No), Horse (Yes, No), Goats (Yes, No), Sheep (Yes, No), Chicken (Yes, No), Pigs (Yes, No), Rabbits (Yes, No), Truck (Yes, No), Ducks (Yes, No), Motor boat (Yes, No), Boat (Yes, No), Sofa set (Yes, No), cupboard (Yes, No) and Cook using (Open fire, open stove).

The above 37 socioeconomic indicator variables were loaded and run using Principal Component Analysis (PCA) to produce the components, eigenvalues, proportion and cumulative proportions plus eigenvectors. We then rotated factor 1 using orthogonal varimax (Kaiser off) method to produce component, variance, difference, proportion, cumulative proportions, rotated components and component rotation matrix for all the variables. To predict socioeconomic status, we scored the coefficients for orthogonal varimax rotation sum of squares (column loading) = 1 for all the variables and then generated socioeconomic status which is linear variable together with its frequency, percentage and cumulative percentage. To generate quintiles, we categorise the socioeconomic status using the cumulative percentage. The first quintile started from minimum up to 20% point on the cumulative percentage, the second quintile starts from 21% up to 40%, the third quintile starts from 41% up to 60%, fourth quintile starts from 61% up to 80% and the fifth quintile starts from 81% up to 100%. The lowest 20% of households were classified as the poorest according to the economic status variable and the highest 20% as the least poor household [18]. We adjusted for data clustering at the health facility level.

## Results

A total of 120 health facilities across Uganda were included in the study; 30 COBERS sites and 90 non-COBERS sites. As Table 1 shows, the COBERS and non-COBERS sites are not significantly different regarding rural-urban location, health facility level and location with respect to malaria risk zoning.

A total of 2399 households were interviewed with 600 households in the COBERS catchment areas and 1799 households in the non-COBERS areas. As Table 2 below shows, the households in the communities of the COBERS and non-COBERS sites are similar with respect to household location, size, sex and age distribution, including the proportion of children under five years of age in the respective populations and wealth quintiles.

**Table 2:** Demographic characteristics of COBERS and Non-COBERS Households

Characteristics	COBERS		Non-COBERS	
Household location	n	%	n	%
Rural	538	90.0	1469	82.0
Urban	62	10.0	330	18.0
Household population				
Rural	3034	90.0	8289	84.3
Urban	336	10.0	1546	15.7
Sex				
Male	1611	48.5	4685	48.4
Female	1709	51.5	4973	51.6
Age groups (years)				
0–20	1983	62.7	5804	62.8
21–40	997	31.5	2855	30.9
41–60	150	4.7	503	5.4
> 60	34	1.1	78	0.8
Population of under 5 years	783	20.5	2513	22.9
Wealth quintile of households				
Poorest Quintile	111	21	305	20
2nd Quintile	99	18	319	21
3rd Quintile	115	21	302	19
4th Quintile	123	23	294	19
Least Poor Quintile	93	17	325	21

A total of 1451 (58%) of under 5 years children in non-COBERS sites used ITN while 523 (67%) of children under 5 years in COBERS communities used ITN (AOR = 0.66, 95% CI 0.47–0.93 & *P*-Value = 0.017). Table 3 summarises the results for factors associated with ITN use.

**Table 3:** Adjusted and Unadjusted O.R for Children under 5 years who slept under ITN the night before survey

Background characteristics	Numerator	Denominator	%	OR	95% CI	p-value	AOR	95% CI	p-value
Site status									
COBERS	523	783	66.8	1			1		
Non-COBERS	1451	2513	57.8	0.77	0.64–0.93	0.06	0.66	0.47–0.93	0.017
Sex									
Female	937	1579	59	1			1		
Male	998	1648	61	1.06	0.91–1.25	0.453	1.05	0.89–1.24	0.579
Sector									
Rural	1638	2819	58	1			1		
Urban	336	477	70	2.37	1.81–3.09	< 0.001	1.9	1.23–2.94	0.004
Wealth Quintile									
Poorest Quintile	270	496	54	1			1		
2nd Quintile	352	669	53	1.16	1.02–1.31	0.02	1.06	0.79–1.43	0.691
3rd Quintile	523	895	58	1.44	1.28–1.63	< 0.001	1.45	1.02–2.05	0.039
4th Quintile	329	521	63	1.89	1.65–2.16	< 0.001	1.65	1.12–2.43	0.012
Least Poor Quintile	328	420	78	3.98	3.38–4.69	< 0.001	3.62	2.29–5.74	< 0.001

*OR* Odds Ration, *AOR* Adjusted Odds Ratio

One hundred and sixty-seven (21%) of the children under 5 years in the COBERS communities had fever in the last two weeks before survey compared to 566 (23%) in the non-COBERS communities. Table 4 summarises the results for factors associated with having fever in the last two weeks before the survey.

**Table 4:** Adjusted and Unadjusted O.R for under 5 children with fever in the last two weeks before survey

Background characteristics	Numerator	Denominator	%	OR	95% CI	p-value	AOR	95% CI	p-value
Site status									
COBERS	167	783	21	1			1		
Non-COBERS	566	2513	23	1.16	0.95–1.41	0.149	1.05	0.68–1.62	0.821
Sex									
Female	364	1579	23	1			1		
Male	357	1648	22	0.92	0.78–1.10	0.361	0.91	0.77–1.07	0.269
Sector									
Rural	612	2819	22	1			1		
Urban	121	477	25	1.28	1.01–1.61	0.041	1.36	0.74–2.52	0.32
Wealth Quintile									
Poorest Quintile	127	496	26	1			1		
2nd Quintile	148	669	22	0.9	0.68–1.20	0.482	0.86	0.60–1.22	0.389
3rd Quintile	197	895	22	0.92	0.71–1.19	0.523	0.89	0.65–1.21	0.448
4th Quintile	110	521	21	0.89	0.66–1.20	0.453	0.84	0.55–1.27	0.398
Least Poor Quintile	86	420	20	0.81	0.59–1.11	0.197	0.7	0.43–1.15	0.158
ITN use by under 5 children									
No	212	903	23	1			1		
Yes	505	1974	26	1.26	1.13–1.41	< 0.001	1.19	0.92–1.54	0.186

*OR* Odds Ration, *AOR* Adjusted Odds Ratio

One hundred (60%) of under 5 children who had fever in the COBERS sites sought care within one day of fever onset compared to 268 (47%) in non COBERS communities (AOR = 0.71, 95% CI 0.55–0.91 and *P*-value = 0.009). Table 5 summarises results for factors associated with seeking care within one day of fever onset.

**Table 5:** Adjusted and Unadjusted O.R for under 5 Children with fever in the two weeks before the survey for whom care was sought within one day

Background characteristics	Numerator	Denominator	%	OR	95% CI	p-value	AOR	95% CI	p-value
Site status									
COBERS	100	167	60	1			1		
Non-COBERS	268	566	47	0.75	0.58–0.96	0.023	0.71	0.55–0.91	0.009
Sex									
Female	182	364	50	1			1		
Male	180	357	50	1.04	0.83–1.28	0.745	1.03	0.83–1.29	0.765
Sector									
Rural	298	612	49	1			1		
Urban	70	121	58	1.53	1.13–2.08	0.007	1.71	1.21–2.41	0.002
Wealth Quintile									
Poorest Quintile	59	127	46	1			1		
2nd Quintile	72	148	49	0.78	0.55–1.10	0.157	0.73	0.52–1.04	0.084
3rd Quintile	93	197	47	0.96	0.70–1.34	0.83	0.91	0.65–1.27	0.591
4th Quintile	65	110	59	1.38	0.95–2.00	0.089	1.28	0.88–1.87	0.192
Least Poor Quintile	44	86	51	0.82	0.54–1.22	0.333	0.68	0.45–1.05	0.082

*OR* Odds Ration, *AOR* Adjusted Odds Ratio

Also, 115 (69%) of children with fever in the COBERS communities took antimalarial drug within 24 h of fever onset compared to 360 (64%) in the non COBERS communities. Table 6 summarises the results for factors associated with taking antimalarial drug within 24 h of fever onset.

**Table 6:** Adjusted and Unadjusted O.R for under 5 years Children with fever in the two weeks before the survey who were given antimalarial drugs within one day

Background characteristics	Numerator	Denominator	%	OR	95% CI	p-value	AOR	95% CI	p-value
Site status									
COBERS	115	167	69	1			1		
Non-COBERS	360	566	64	0.77	0.53–1.10	0.15	0.6	0.27–1.32	0.204
Sex									
Female	236	364	65	1			1		
Male	229	357	64	0.93	0.69–1.26	0.658	0.85	0.48–1.48	0.558
Sector									
Rural	392	612	64	1			1		
Urban	83	121	69	1.28	0.82–2.00	0.275	1.38	0.64–2.99	0.409
Wealth Quintile									
Poorest Quintile	80	127	63	1			1		
2nd Quintile	93	148	63	0.93	0.58–1.50	0.781	0.62	0.25–1.54	0.297
3rd Quintile	121	197	61	1.24	0.78–1.97	0.365	1.44	0.56–3.70	0.449
4th Quintile	72	110	65	0.98	0.60–1.61	0.936	0.92	0.35–2.42	0.858
Least Poor Quintile	66	86	77	1.27	0.71–2.28	0.428	1.87	0.61–5.79	0.274

*OR* Odds Ration, *AOR* Adjusted Odds Ratio

## Discussion

This study shows significant differences between COBERS and non-COBERS sites. Children under 5 years at COBERS sites were more likely to use preventive and curative measures for malaria, although there was no difference in the prevalence of fever. This finding is consistent with that of a study done in Nigeria which showed that community-based medical training increased community awareness of preventive aspects for various communicable and non-communicable diseases [16]. This study also shows differences in ITN use between urban and rural children with urban children more likely to sleep under ITN than rural children. In our study, 70% of children in urban compare with 58% in rural areas slept under ITN the night before the survey. However, this result is in contrast with the UMIS 2014–15 which shows that 71.5 of children under 5 years in urban areas slept under ITN compare with 75% of children under 5 years in rural areas [8]. Also, children in higher wealth quintile were more likely to use ITN than children from low wealth quintiles. Given that socio-economic status is a major determinant of the ownership and use of ITN among children under 5 years [4, 19], it is important to note that there is no difference in socio-economic status between the households in the COBERS and non- COBERS communities. Thus, socio-economic status does not account for the observed difference in the use of ITNs by under-5-year children between the COBERS and non-COBERS sites. The difference could be attributed to interaction with medical students.

There was no statistical difference between COBERS and non-COBERS sites regarding children under 5 years experiencing fever. However, there was statistical significant difference between COBERS communities and non-COBERS communities regarding children under 5 years who sought care within the recommended time frame of 24 h (AOR = 0.71, *p* = 0.009).

Students who are placed for COBERS get involved in PHC activities like community mobilisation, home and school visits and health educational talks. Research findings from other studies suggest that community members reported that they have seen improvements in health and health seeking behaviours and increased community participation in PHC because of CBE [16]. PHC is intended to address the major local health problems of a country population and it is the interface between the community and the health system [20].

Also, important to note is that a greater number of children received a drug (antimalarial or otherwise) within the recommended time frame of 24 h both in the COBERS and non COBERS communities compared to those who sought care in both COBERS and non-COBERS communities. Taken together, these findings suggest that there is possible self-medication or medication in the home. Plausibly, this could be attributed to inherence drug stock out at the health facilities in Uganda as has been noted in the annual health sector performance report [6]. However, there was a statistically significant difference between the COBERS and non-COBERS sites in seeking care within one day for children under 5 years of age. Thus, although there is possibly medication taking place in the home in both COBERS and non-COBERS communities, the COBERS communities are more likely to seek care for children under five with fever than the non-COBERS communities. Other studies have shown that several African countries were far from achieving the RBM set targets of 80% [10, 11, 21]. RBM aimed at ensuring that those suffering from malaria have prompt access to and able to correctly use affordable and appropriate treatment within 24 h of symptom onset [9].

### Study limitation

There are some limitations to consider while interpreting our findings. We did not test those who reported to be having fever for presence of malaria parasites but rather took all persons who reported to have had febrile illness as malaria. This was subjective because they were based on head of household or mother perception of illness and were not necessarily validated by medical personnel thus it may not be true that all febrile illness is malaria. Therefore, we could have overestimated the incidence of malaria in the study population. Secondly, we can not discount social desirability bias in our study since most parents or household heads may want to show that they are complying with government program. Thirdly, the cross-sectional design makes it difficult to determine whether the observed outcomes are because of exposure to COBERS. However, despite this, our study has shown that COBERS training is associated with improve malaria prevention through sleeping under ITNs and seeking treatment for fever within the recommended time frame of 24 h for children under 5 years in Uganda. Besides, our study is the first of its kind to be conducted in Uganda to assess COBERS contribution to community health.

## Conclusion

This study provides some evidence to suggest that COBERS training has influenced health behaviours in the communities where students are placed for preventive care for children with malaria. Further studies are needed to assess the causal relationship, and the longer-term impact of COBERS on community health and other objectives of the COBERS program in Uganda.

## Additional file

Additional file 1: Household Questionnaire used for the baseline collection of data during survey. (PDF 136 kb)

## Abbreviations

AOR: Adjusted Odds Ratio
CBE: Community-Based Education
COBERS: Community-Based Education Research and Service
COR: Crude Odds Ration
IRS: Indoor Residual Spraying
ITNs: Insecticide-Treated Nets
MEPI: Medical Education Partnership Initiative
MESAU: Medical Education for Equitable Services to All Ugandans
RBM: Roll Back Malaria
UDHS: Uganda Demographic and Health Survey
UMIS: Uganda Malaria Indicator Survey
