# Supplementary material for: Community-based training of medical students is associated with malaria prevention and treatment seeking behaviour for children under 5 years in Uganda: a study of MESAU-MEPI COBERS in Uganda
Source: BMC Med Educ. 2018 Jun 8;18:131. doi: 10.1186/s12909-018-1250-y (PMC5994002; doi:10.1186/s12909-018-1250-y)
Supplement: Supplementary file 1 — Household Questionnaire used for the baseline collection of data during survey. (PDF 136 kb) [file 12909_2018_1250_MOESM1_ESM.pdf]

Medical Education for Equitable Services to All Ugandans (MESAU) COBERS Evaluation Household Survey

Household Questionnaire

**INFORMATION PANEL**		
Fill in the village and household number,<br>*Fill in your name, number and the date.*		
HH1. District Name:		HH2: COBERS Site Health Facility Name:
HH3. Village number:		HH4. Sector: Rural……….1 Urban……..……….. 2
HH5. Household number:		
HH6. Day/Month/Year of interview (dd/mm/yyyy):		
HH7. Interviewer name:		HH8: Interviewer code:
HH9. Result of interview	Completed ....1<br>Not at home ....2<br>Refused ....3<br>Partly completed ....4<br>Incapacitated ....5<br><br>Other (*specify*) ________________________________ 6	
HH10. CONTINUATION SHEET ☐		

INTRODUCTION AND CONSENT

Hello. My name is…………………………………………………. and I am working with [MESAU or specific MESAU institution]. We are conducting a survey to find out whether our students have an impact on the community through COBERS. We would very much appreciate your participation in this survey. The survey usually takes about 30 to 45 minutes to complete.

As part of the survey we would first like to ask some questions about your household. All of the answers you give will be confidential. If we should come to any question you don't want to answer, just let me know and I will go on to the next question. At this time, do you want to ask me anything about the survey?

May I begin the interview now?

Signature of interviewer: ______________________________ Date: ____________________________________

RESPONDENT AGREES TO BE INTERVIEWED . . . 1   RESPONDENT DOES NOT AGREE TO BE INTERVIEWED . . . 2 →END

Signature of Team Leader (sign after checking the form):______________________

PART I. HOUSEHOLD SCHEDULE AND HEALTH SERVICE UTILIZATION
THIS MODULE IS TO BE ANSWERED BY EITHER 1. HEAD OF HOUSEHOLD OR 2. OTHER ADULT MEMBER OF THE HOUSEHOLD WHO IS KNOWLEDGEABLE ABOUT THE HEALTH OF ALL MEMBERS OF LIVING IN THE HOUSEHOLD

**100**	**101**	**102**	**103**		**104**	**105**	**106**	**107**	**108**
**Line No.**	Please name all the people in the household, starting with the oldest. Don't forget to include yourself.	Is (name) male or female?<br><br>M...... 1<br>F 0	How old is (name) in completed years?<br><br>**IF LESS THAN ONE YEAR, RECORD COMPLETED MONTHS**		What is the highest level of formal education (name) has attended?<br><br>None .... 0<br>Primary .... 1<br>O' Level........... 2<br>A' Level .... 3<br>Tertiary .... 4<br>University .... 5<br><br>DK .... 98<br><br>**IF UNDER 6 YEARS OF AGE**<br>NA……………..998	What is (name's) marital status?<br><br>Never married .... 1<br>Monogamously married ..... 2<br>Polygamously married .... 3<br>Divorced/separated .... 4<br>Widowed .... 5<br>Other .... 7<br>*(Specify)*<br><br>**IF UNDER 15 YEARS OF AGE**<br>NA………………………..998	Is (name) currently employed or earning an income?<br><br>Yes ..... 1<br>No....... 0<br>DK 98<br><br>**IF UNDER 15 YEARS OF AGE**<br>NA…………998	In the past 2 weeks, has (name) been unable to perform normal activities because of any health related reason?<br><br>Yes ..... 1<br><br>**IF ANSWER FOR HOUSEHOLD HEAD IS "NO" OR "DK"**<br>No....... 0➔201<br>DK....... 98 ➔201<br><br>**IF ANSWER FOR OTHER HOUSEHOLD MEMBERS IS "NO" OR "DK"**<br>No....... 0➔301<br>DK....... 98 ➔301	What was the main complaint or illness?<br><br>Fever/malaria ....1<br>Cough/chest infection ...2<br>Tuberculosis ....3<br>Asthma ....4<br>Headache ....5<br>Diarrhea........................6<br>Vomiting........................7<br>Stomach pain................8<br>Skin problem.................9<br>Ear.............................. 10<br>Eye problem .... 11<br>Hypertension .... 12<br>Diabetes .... 13<br>Pregnancy related .... 14<br>Delivery related............ 15<br>HIV/AIDS .... 16<br>Injury .... 17<br>Other............................ 18<br>*(Specify)*
			**Y**	**M**					
01									
02									
03									
04									
05									
06									
07									
08									

**PART I. HOUSEHOLD ROSTER AND HEALTH SERVICE UTILIZATION:** THIS MODULE IS TO BE ANSWERED BY 1. HEAD OF HOUSEHOLD OR 2. OTHER ADULT MEMBER OF THE HOUSEHOLD WHO IS KNOWLEDGEABLE ABOUT THE HEALTH OF ALL MEMBERS OF LIVING IN THE HOUSEHOLD					
**ID Code**	**109**	**110**	**111**	**112**	**113**
**LINE NO.**	Did *(name)* seek care outside the home for this condition?<br><br>Yes….1➔111<br>No…..0➔110<br>DK…98 ➔113<br><br>**IF THE RESPONDENT SAYS "NO", PROBE FOR WHETHER TREATMENT WAS SOUGHT FROM TRADITIONAL HEALERS AND OTHER SOURCES**	What was the main reason that treatment was not sought for *(name)* outside home for his/her condition?<br><br>Too expensive....................................1<br>Too far..2<br>Too busy (work, children)...................3<br>Wasn't sick enough............................4<br>Facility has poor infrastructure...........5<br>Facility poorly stocked........................6<br>Poor staff attitude...............................7<br>Poor staff knowledge..........................8<br>Don't trust the staff.............................9<br>Self-treated only.................................10<br>Other (specify) ....11<br><br>**DO NOT READ THE OPTIONS ALOUD. RECORD ONLY ONE RESPONSE. MOVE ON TO QN. 113 AFTER COMPLETING THIS QUESTION.**	Where did *(name)* seek care outside home for this condition?<br><br>Government hospital................................1<br>Government HCIV...................................2<br>Government HCIII...................................3<br>Government II...4<br>Private hospital.......................................5<br>Private clinic/health post..........................6<br>Medical personnel...................................7<br>Traditional healer....................................8<br>Faith/Church healer.................................9<br>Community health worker*.......................10<br>Pharmacy/drug seller...............................11<br>Other (specify) ....12<br><br>*Village Health Team member<br>**DO NOT READ THE OPTIONS ALOUD. IF HOSPITAL, CLINIC OR CENTER PROBE FOR NAME OF FACILITY**	How far is your household to this point of service? *(One way in Kilometers)*	How far is your household from the nearest health care facility (HCII, III, IV, hospital)? (One way in Kilometers)<br><br>**RECORD RESPONSE IN THE SPACE PROVIDED ABOVE**
01					
02					
03					
04					
05					
06					
07					
08					

PART II. HOUSEHOLD CHARACTERISTICS MODULE		
201. What is the religion of the head of this household?	Catholic....1<br>Church of Uganda ....2<br>Islam ....3<br>Traditional....4<br>No religion ....5<br>Other (Specify)....6	
202. Ethnic group of head of household	Ethnic Group ______________________________	
203. How many rooms in this household are used for sleeping?	No. of rooms __ __	
204. Main material of the dwelling floor:<br><br>**RECORD OBSERVATION.**	Natural floor<br>Earth/sand ....1<br>Earth and Dung ....2<br><br>Finished floor<br>Parquet or polished wood....3<br>Mosaic or tiles....4<br>Bricks....5<br>Cement....6<br>Stones ....7<br>Other (*specify*) ____________________________ 96	
205. Main material of the roof.<br><br>**RECORD OBSERVATION.**	Natural roofing<br>Thatched....1<br>Mud....2<br><br>Finished roofing<br>Iron sheets....3<br>Wood/planks....4<br>Asbestos....5<br>Tiles....6<br>Tin....7<br>Cement....8<br>Other (*specify*) ____________________________ 96	
206. Main material of the walls.<br><br>**RECORD OBSERVATION.**	Natural walls<br>Cane/palm/trunks ....1<br><br>Rudimentary walls<br>Mud and poles....2<br>Un-burnt bricks ....3<br>Un-burnt bricks with plaster....4<br>Burnt bricks with mud ....5<br><br>Finished walls<br>Cement blocks....6<br>Stone ....7<br>Burnt bricks with cement ....8<br>Timber ....9<br>Other (specify) ____________________________ 96	

207. What type of fuel does your household mainly use for cooking?	Electricity ....1<br>LPG/Natural Gas ....2<br>Biogas....3<br>Kerosene/paraffin ....4<br>Charcoal ....5<br>Firewood....6<br>Straw/shrubs/grass....7<br>Animal dung....8<br>No food cooked in household ....11<br>Other (*specify*)_______________ 96	
208a. In this household, is food cooked on an open fire or a stove?	Open fire....1<br>Open stove ....2<br>Other (*specify*) _______________6➔**209**	
208b. Does the fire/stove have a chimney?	Yes ....1<br>No....2	
209a. Is the cooking usually done in the house, in a separate building, or outdoors?	In the house....1<br>In a separate builidng ....2<br>Outdoors....3<br>Other (*specify*) _______________ 6	
209b. Do you have a separate room which is used as a kitchen?	Yes ....01<br>No....02	
210. Does your household have:	Yes No<br>Electricity .... 1 2<br>Radio .... 1 2<br>Cassette player.... 1 2<br>Television/VCR/DVD/VCD.... 1 2<br>Mobile phone .... 1 2<br>A fixed telephone.... 1 2<br>Refrigerator.... 1 2<br>Table.... 1 2<br>Chair .... 1 2<br>Sofa set .... 1 2<br>Bed .... 1 2<br>Cupboard Table.... 1 2<br>Clock.... 1 2	
211. Does any household member own:	Yes No<br>Bicycle .... 1 2<br>Motorcycle/Scooter .... 1 2<br>Animal drawn-cart.... 1 2<br>Car/Truck.... 1 2<br>Boat with motor.... 1 2<br>Boat with no motor.... 1 2	

212. Does any member of this household own any land that can be used for agriculture?	Yes ....1<br>No................................2➔214	
213 Type of ownership	Customary ....01<br>Communal ....02<br>Mailo ....03<br>Freehold ....04<br>Leasehold 05	
214. How many of the following animals does this household have?<br>IF NONE, RECORD '00'.<br>IF MORE THAN 95, RECORD '95'.<br>IF UNKNOWN, RECORD '98'.<br><br>i) Local cattle?<br>ii) Exotic/cross cattle?<br>iii) Horses, donkeys, or mules?<br>iv) Goats?<br>v) Sheep?<br>vi) Chicken?<br>vii) Pigs?<br>viii) Rabbits<br>ix) Duck	 <br><br><br><br>Local cattle ___ ___<br>Exotic/cross cattle ___ ___<br>Horses, donkeys, or mules ___ ___<br>Goats ___ ___<br>Sheep ___ ___<br>Chicken ___ ___<br>Pigs ___ ___<br>Rabbits ___ ___<br>Ducks ___ ___	

215 HOUSEHOLD MEMBERS WHO HAVE DIED WITHIN THE LAST 12 MONTHS<br><br>215a Think back over the past 12 months. Has any usual member of household died in the last 12 months?<br><br>215b How many household members died in the last 12 months?			Yes……………………………………..1<br>No……………………………………….2 ➔301<br>Don't know ……………………………..8<br><br>Number of deaths………………	
Ask the next questions for each person who died				
215c What was the name of the person who died (most recently/before him/her)	Name of 1st death<br>________________________	Name of 2nd death<br>____________________	Name of 3rd death<br>____________________	Name of 3rd death<br>____________________
215 Was (NAME) male or female?	Male………………………1<br>Female……………………2	Male………………………1<br>Female……………………2	Male………………………1<br>Female……………………2	Male………………………1<br>Female……………………2
215e How old was (NAME) when (he/she) died?<br><br>**COMPLETED YEARS (OR COMPLETED MONTHS IF UNDER 1 YEAR)**	Age……………..	Age…………….	Age…………..	Age…………..
215f What was the cause of (NAME'S) death?<br><br>**CIRCLE THE NUMBER CORREPONDING TO THE CAUSE OF DEATH**<br><br>**MULTIPLE RESPONSES ALLOWED**	Fever/malaria ....A<br>Cough/chest infection...B<br>Tuberculosis................C<br>Asthma ....D<br>Headache....................E<br>Diarrhea.......................F<br>Vomiting ....G<br>Stomach pain ....H<br>Hypertension ....I<br>Diabetes ....J<br>Pregnancy related ....K<br>Delivery related ....L<br>HIV/AIDS.....................M<br>Injury............................N<br>Other ....X<br>*(Specify)*	Fever/malaria ....A<br>Cough/chest infection...B<br>Tuberculosis................C<br>Asthma ....D<br>Headache....................E<br>Diarrhea ....F<br>Vomiting ....G<br>Stomach pain ....H<br>Hypertension ....I<br>Diabetes ....J<br>Pregnancy related ....K<br>Delivery related ....L<br>HIV/AIDS.....................M<br>Injury ....N<br>Other ....X<br>*(Specify)*	Fever/malaria ....A<br>Cough/chest infection...B<br>Tuberculosis................C<br>Asthma ....D<br>Headache....................E<br>Diarrhea ....F<br>Vomiting ....G<br>Stomach pain ....H<br>Hypertension ....I<br>Diabetes ....J<br>Pregnancy related ....K<br>Delivery related ....L<br>HIV/AIDS.....................M<br>Injury ....N<br>Other ....X<br>*(Specify)*	Fever/malaria ....A<br>Cough/chest infection...B<br>Tuberculosis................C<br>Asthma ....D<br>Headache....................E<br>Diarrhea ....F<br>Vomiting ....G<br>Stomach pain ....H<br>Hypertension ....I<br>Diabetes ....J<br>Pregnancy related ....K<br>Delivery related ....L<br>HIV/AIDS.....................M<br>Injury ....N<br>Other ....X<br>*(Specify)*

PART III MALARIA

THIS MODULE IS TO BE ANSWERED BY EITHER 1. HEAD OF HOUSEHOLD OR 2. OTHER ADULT MEMBER OF THE HOUSEHOLD WHO IS KNOWLEDGEABLE ABOUT THE HEALTH OF ALL MEMBERS OF LIVING IN THE HOUSEHOLD

ID	301	302	303	304	305	306	307	308	309
**Line No.**	In the last two weeks, that is, since (*day of the week*) of the week before last, has (*name*) been ill with a fever?<br><br>Yes...1<br>No….2 ➔306<br>DK…98 ➔306	Was (*name*) seen at a health facility or by any health worker during this illness?<br><br>Yes...1<br>No….2➔306<br>DK…98➔306	How many days after fever started was (name) taken to the facility?<br><br>**RECORD "0" IF TAKEN ON THE SAME DAY WHEN FEVER STARTED**	Was (*name*) given medicine for malaria by the health worker or at the health facility?<br><br>Yes...1<br>No….2➔306<br>DK…98➔306	How long after the fever started did (*name*) first take the anti-malarial?<br><br>**IF SAME DAY, RECORD 0.**	Did (*name*) sleep under an Insecticide Treated mosquito net last night?<br><br>Yes...1<br>No….2 ➔401<br>DK…98	What type of Insecticide Treated mosquito net is this?<br><br>Long lasting treated net……1<br>Re-treatable net…………… 2<br>OTHER (specify)………..… 3<br>DK…………………………..98<br><br>**IF THE RESPONDENT DOES NOT KNOW THE TYPE OF THE NET, IF POSSIBLE, OBSERVE THE NET.**	Since you got the mosquito net, was it ever soaked or dipped in a liquid to kill/repel mosquitoes or bugs?<br><br>Yes...1<br>No….2<br>DK…98	How long ago was the net last soaked or dipped?<br><br>**RECORD MONTHS. IF MORE THAN A YEAR, RECORD 95.**
01									
02									
03									
04									
05									
06									
07									
08									

PART IV. COBERS EXPOSURE

401a Has anyone in this household ever seen health professional students of *(institution)* at *(name of health facility)*?	Yes…………………………………………….1<br>No ..……………………………………………2 ➔402<br>DK……………………………………………..98
401b. What activities were the student doing?<br><br>**MULTIPLE RESPONSES ALLOWED**	Treating patients…………………………..A<br>Giving immunizations………………………B<br>Giving health education talks....................C<br>Filling out forms……………………………D<br>Other (specify) _______________________________
402a. Has anyone in this household ever seen health professional students of *(institution)* in this community?	Yes…………………………………………….1<br>No ..……………………………………………2 ➔403<br>DK……………………………………………..98
402b. What activities was the student doing?<br>**MULTIPLE RESPONSES ALLOWED**	Giving immunizations……………………….A<br>Giving health education talks……………..B<br>Distributing bed nets………………………..C<br>Home visit……………………………………D<br>Interviewing people………………………….E<br>Other (specify)______________________________
403. Has anyone in this household ever interacted with a University student who is in training for a health profession in this community?	Yes…………………………………………….1<br>No ..……………………………………………2 ➔405<br>DK……………………………………………..98 ➔405

404. ***[For respondents who have interacted with a health student]*** I am going to ask you how much you agree or disagree with the following statements about University students who are in the health professions *(Mark in appropriate column)*

**Statement**	Strongly Disagree	Disagree	Agree	Strongly Agree
404a. Students can bring new ways of thinking about health problems in my community.	1	2	3	4
404b. Students can help my community solve its health problems.	1	2	3	4
404c. Students can make health services more available in my community.	1	2	3	4
404d. Students are welcome in this community.	1	2	3	4
404e. When students are at the health facility, I spend less time waiting.	1	2	3	4
404f. Students do not stay in the community long enough to make a difference.	1	2	3	4
404g. Students behave in a respectful way.	1	2	3	4

405. ***[For respondents who have not interacted with a health student]*** I am going to ask you how much you agree or disagree with the following statements about University students who are in the health professions *(Mark in appropriate column)*

**Statement**	Strongly Disagree	Disagree	Agree	Strongly Agree
405a. Students can bring new ways of thinking about health problems in my community	1	2	3	4
405b. Students can help my community solve its health problems	1	2	3	4
405c. Students can make health services more available in my community.	1	2	3	4
405d. Students are welcome in this community.	1	2	3	4
